# The relationship between vitamin K and metabolic dysfunction-associated fatty liver disease among the United States population: National Health and Nutrition Examination Survey 2017–2018

**DOI:** 10.3389/fnut.2023.1086477

**Published:** 2023-04-14

**Authors:** Xinyue Wang, Wei Zhang, Jiale Huang, Hongwei Li, Jian Gao

**Affiliations:** ^1^Department of Nutrition, Xiamen Clinical Research Center for Cancer Therapy, Zhongshan Hospital (Xiamen), Fudan University, Xiamen, Fujian province, China; ^2^School of Public Health, Xiamen University, Xiamen, China; ^3^Department of Clinical Nutrition, The First Affiliated Hospital of Nanchang University, Nanchang, Jiangxi, China; ^4^Department of Nutrition, Zhongshan Hospital, Fudan University, Shanghai, China

**Keywords:** vitamin K, metabolic dysfunction-associated fatty liver disease, dietary supplements, NHANES, cross-sectional analysis

## Abstract

**Background:**

The effect of vitamin K is associated with several pathological processes in fatty liver. However, the association between vitamin K levels and metabolic dysfunction-associated fatty liver disease (MAFLD) remains unclear.

**Objective:**

Here, we investigated the relationship between vitamin K intake and MAFLD risk by employing the American National Health and Nutrition Examination Surveys (NHANES) including 3,571 participants.

**Methods:**

MAFLD was defined as hepatic steatosis with one or more of the following: overweight or obesity, type 2 diabetes, or >2 other metabolic risk abnormalities. The total vitamin K was the sum of dietary and supplement dietary intake. The relationship of between log_10_(vitamin K) and MAFLD was investigated using survey-weighted logistic regression and stratified analysis, with or without dietary supplementation.

**Results:**

The MAFLD population had a lower vitamin K intake than the non-MAFLD population (*p* = 0.024). Vitamin K levels were inversely associated with MAFLD in the fully adjusted model (OR = 0.488, 95% CI: 0.302–0.787, *p* = 0.006). Consistent results were seen in the group without dietary supplements (OR = 0.373, 95% CI: 0.186–0.751, *p* = 0.009) but not in the group consuming dietary supplements (OR = 0.489, 95% CI: 0.238–1.001, *p* = 0.050).

**Conclusion:**

Vitamin K intake may be a protective factor for MAFLD, especially for individual not using dietary supplements. Nevertheless, more high-quality prospective studies are needed to clarify the causal relationship between them.

## 1. Introduction

Fatty liver is a rapidly progressive chronic liver disease with an estimated global prevalence of 24% (1) and approximately 1/3 of adults in the United States have a fatty liver (2). Nonalcoholic fatty liver disease (NAFLD) is the hepatic manifestation of metabolic syndrome, a spectrum of diseases ranging from benign hepatic steatosis to nonalcoholic steatohepatitis that may progress to cirrhosis and liver cancer (3). In 2020, a classification of metabolic dysfunction-associated fatty liver disease (MAFLD) was proposed based on the diagnosis of hepatic steatosis, while incorporating other markers of metabolic abnormalities such as insulin-resistance, high-sensitivity reactive protein, and other metabolic risk factors for pathological progression (4, 5). Ultrasound is a pragmatic and widely accepted first-line examination that has good sensitivity (85%) and specificity (95%) in identifying moderate and severe steatosis. Since liver fibrosis may increase liver echogenicity, the presence of underlying chronic liver disease reduces the accuracy of liver fat assessment. To overcome the limitations of ultrasound in assessing low hepatic steatosis levels, more advanced ultrasound techniques have been developed. Controlled Attenuation Parameter (CAP), available on the FibroScan system (Echosens, France), measures the attenuation of the United States beam (6). CAP uses ultrasound and vibration-controlled elastography to measure the ultrasound attenuation degree due to liver fat (7). Meanwhile, owing to the lack of effective therapeutics and efficient policies to evaluate the prevalence of MAFLD, the economic burden of healthcare in the United States is expected to increase. It is important to define appropriate interventions and prevent serious complications regarding MAFLD.

In recent years, studies have shown that the formation and development of fatty liver are a combination of multiple factors that ultimately lead to liver damage, including insulin resistance, adipokines secretion, oxidative stress, lipid peroxidation, mitochondrial damage, endoplasmic reticulum stress, gut microbiota, innate immunity, and genetic and epigenetic mechanisms (8–10). On the other hand, vitamins are micronutrients vital to health and they have previously been identified as new potential targets for indirect therapy for MAFLD (11). Several studies have linked liver disease to vitamin deficiencies, and vitamin supplementation may protect liver tissue by reducing insulin resistance, lipid peroxidation, and fatty acid synthesis, and improving hepatic steatosis (12–14). Studies have shown that vitamin K deficiency occurs in many pathological conditions (e.g., liver disease, cholestasis, cystic fibrosis, alcoholism, malabsorptive states, and bariatric surgical interventions) (15, 16), and that vitamin K supplementation affects the immune system, anti-inflammation, gut microbes and their metabolites, antioxidants and coagulation, and epithelial development (17, 18). These effects are associated with several pathological processes in fatty liver; therefore, vitamin K may have a protective effect against the occurrence and progression of MAFLD.

Little is known about the role of vitamin K in lipid metabolism. Although one study described a positive association between concentrations in adipose tissue (19), studies exploring the association between vitamin K and MAFLD are lacking, and the association between vitamin K and MAFLD remains unexplored. Therefore, in the current study, we investigated the relationship between vitamin K levels and the risk of MAFLD by employing the American National Health and Nutrition Examination Surveys (NHANES).

## 2. Materials and methods

### 2.1. Data source and study sample

The data were collected by the United States Centers for Disease Control and Prevention (CDC) using a stratified, multistage, and probability-cluster design. The Ethics Review Board of the National Center for Health Statistics approved the NHANES protocol and informed consent was obtained from all participants (20). All data can be freely downloaded from the NHANES website.^1^ NHANES 2017–2018 is the only publicly available survey database for liver fibrosis assessment by FibroScan® and it has been used in studies of MAFLD (21). The initial sample size was 9,254 people from 2017 to 2018. We excluded subjects younger than 20 years old (*n* = 3,685), viral hepatitis (*n* = 50), pregnancy (*n* = 54), the incomplete diagnostic indicators for MAFLD (*n* = 489), and missing a mean of two 24 h recall dietary data for vitamin K (*n* = 1,040), body mass index (BMI, *n* = 51), alanine aminotransferase (ALT, *n* = 260), aspartate aminotransferase (AST, *n* = 13), C-reactive protein (CRP, *n* = 12), and minutes sedentary activity (*n* = 29). Finally, 3,571 participants were included in this study. The details are shown in Supplementary Figure S1.

### 2.2. Dietary intake data and supplement dietary intake date

Dietary intake data and dietary supplement intake were obtained from two 24 h-recall interviews with NHANES. The first interview was arranged face-to-face at the Mobile Examination Center (MEC) and the second interview was conducted by telephone 3–10 days later. Energy and nutrient intake for each food or beverage were calculated using the Food and Nutrient Database for Dietary Studies (FNDDS). In this study, dietary intake and dietary supplement intake were estimated using the mean of two 24-recall data points, and the total energy and nutrients were the sum of the dietary intake and dietary supplement intake. Another, study showed that was no significant difference in the energy intake reported in the first and second interviews (22), therefore, this was considered a good intake dataset to determine the average dietary intake for each individual.

### 2.3. Diagnosis of MAFLD

MAFLD was defined as hepatic steatosis with one or more of the following: (1) overweight or obesity (body mass index ≥ 25 kg/m^2^); (2) type 2 diabetes; or (3) two or more other metabolic risk abnormalities: (1) blood pressure ≥ 130/85 mmHg or specific drug treatment; (2) overweight or obesity (body mass index ≥ 25 kg/m^2^); (3) plasma high-density lipoprotein-cholesterol < 40 mg/dL for men and <50 mg/dL for women or specific drug treatment; (4) plasma triglycerides ≥ 150 mg/dL or specific drug; (5) homeostasis model assessment of insulin resistance score ≥ 2.5; (6) prediabetes (fasting glucose 100–125 mg/dL or hemoglobin A1c (HA1c) 5.7%–6.4%); and (7) plasma CPR level > 2 mg/L (21).

Hepatic steatosis was defined by CAP, and the steatosis was stratified as S0-S3. The thresholds of CAP for S1-S3 were 248, 268, and 280, respectively (6). Hepatic fibrosis was defined by liver stiffness measurements, and the stiffness was stratified as F1-4, and the LSM for F1-4 were 6.3, 8.3, 10.5, and 12.5, respectively (23). Significant steatosis and stiffness were diagnosed as a grade greater than S1 and F1. In this study, participants with a fasting time of <3 h, less than 10 complete LSM readings, or a liver stiffness interquartile (IQR) range/median LSM of more than 30% were deemed to have failed FibroScan® measurements and were excluded (21).

### 2.4. Other covariates

#### 2.4.1. Demographic characteristics

Self-reported demographic variables included age (years), sex (men/women), race (Mexican American, other Hispanic, non-Hispanic white, non-Hispanic black, non-Hispanic Asian, or other races, including multiracial), and educational level (<11th grade, high school graduate, some college education, college graduate, or above).

#### 2.4.2. Body measurement

Trained health technicians obtained various body measurements including height, and weight at the MEC. BMI was calculated using weight and height information. The formula used is as follows:


$$BMI\left(\frac{kg}{m^{2}}\right)=weight\left(kg\right)÷height^{2}\left(m\right)$$

#### 2.4.3. Biochemical indicators

Serum samples were processed, stored, and shipped to the University of Minnesota Advanced Research Diagnostic Laboratory (ARDL) in Minneapolis for analysis (24). Regarding biochemical indicators, AST, ALT, TC (cholesterol), TG (triglycerides), LDL (low-density lipoprotein), HDL (high-density lipoprotein), and CRP were included in the regression analysis as covariates.

#### 2.4.4. Lifestyle

The Smoking-Cigarette Use (variable name prefix SMQ) dataset provides a history of cigarette use. Smoking status was classified as never smoker (never smoked 100 cigarettes in a lifetime), some days, or every day Alcohol use was assessed using the dietary interview Total Nutrient Intakes, First Day (DR1TOT_J). Minute sedentary activity was assessed using the Physical Activity Questionnaire (variable name prefix PAQ). The Global Physical Activity Questionnaire (GPAQ) provides respondent-level interview data on physical activity.

### 2.5. Diseases and medications

The diagnostic criteria for hypertension included: the patient being informed by a doctor that they have hypertension, SBP ≥ 140 mmHg or DBP ≥ 90 mmHg. The diagnostic criteria for diabetes mellitus (DM) are: doctor told you have diabetes, or fasting glucose (mmol/l) ≥7.0, or random blood glucose (mmol/L) ≥ 11.1, or glycohemoglobin HbA1c (%) > 6.5, or 2-h OGTT blood glucose (mmol/L) ≥ 11.1, or use of diabetes medication or insulin. Drug information (told to take prescription for cholesterol) used the Blood Pressure/Cholesterol section (variable name prefix BPQ) in the NHANES.

### 2.6. Healthy eating index-2015

Healthy eating index (HEI)-2015 was designed and scored from 0 to 100, which was derived from the sum of 13 components: total fruits, whole fruits, total vegetables, greens and beans, total protein foods, seafood and plant proteins (each 0–5 points); whole grains, dairy, fatty acids, sodium, refined grains, added sugars, and saturated fats (each 0–10 points) (25). A higher HEI-2015 score indicated better diet quality. In this study, the HEI-2015 was calculated using the mean of two 24 h recalls.

### 2.7. Statistical methods

The sampling weights recommended by NHANES for the planned oversampling of specific groups were used in this study. All analyses were sample-weighted and accounted for the complex stratified, multistage, cluster sampling design of NHANES (26, 27). For continuous variables, the survey-weighted median ± standard error was used, and the *p*-value was calculated by the survey-weighted linear regression. For categorical variables, the survey-weighted percentage (standard error) was used, and the *p*-value was calculated by the survey-weighted Chi-square test.

Because vitamin K values had a negatively skewed distribution, they were converted to base 10 log values to conform to a normal distribution. The relationship between log vitamin K and MAFLD was explored using the survey-weighted logistic regression. Model 1 was unadjusted. Model 2 was adjusted for age, sex, and race. Model 3 was adjusted for age, sex, race, education, smoking, alcohol, BMI, CRP, AST, ALT, minutes of sedentary activity, drug, energy, HEI-2015, dietary supplements, drugs, hypertension, and DM. To better explore the association between log vitamin K and MAFLD, multivariable logistic regression was conducted with log vitamin K as the categorical variable, and we divided log vitamin K quartiles. Then, we fitted a linear relationship between log vitamin K and MAFLD by smoothing the curve. Finally, we used stratified logistic regression models for interaction analysis and used restricted cubic splines to estimate the dose–response relationship between log vitamin K intake and MAFLD.

Additional sensitivity analyses were performed to determine: (1) collinearity between vitamin K and other variables by the variance inflation factor (VIF) and (2) extreme vitamin K values that were lower 1% and greater than 99%.

All analyses were performed using R software, version 4.0.3 (R Foundation for Statistical Computing, Vienna, Austria). A two-sided *p*-value < 0.05 was considered significant for all analyses.

## 3. Results

### 3.1. Characteristics in a sex-specific MAFLD population

The general characteristics of the participants are shown in Table 1. The MAFLD population had lower vitamin K intake than the non-MAFLD population (123.113 ± 4.635 vs. 145.064 ± 7.759, *p* = 0.024). Compared with the non-MAFLD population, age, BMI, ALT, CRP, TC, TG, LDL, HDL, energy, the proportion of Mexican Americans, DM, and hypertension were higher in the MAFLD population, while the proportion of higher education was lower.

**Table 1:** Characteristics of participants by categories of MAFLD: NHANES 2017–2018.

Characteristics (weighted)	Total	Non-MAFLD	MAFLD	*p-*value
*N*	3,571	1,479	2,052	
Vitamin K (μg)	132.939 ± 4.519	145.064 ± 7.759	123.113 ± 4.635	0.024
Log vitamin K	1.975 ± 0.014	2.003 ± 0.017	1.952 ± 0.017	0.017
Energy (kcal)	2091.696 ± 21.263	2030.246 ± 25.442	2141.495 ± 27.386	0.004
Age (years)	48.500 ± 0.681	44.229 ± 0.831	51.962 ± 0.699	<0.0001
BMI (kg/m^2^)	29.896 ± 0.294	25.845 ± 0.310	33.178 ± 0.330	<0.0001
ALT (u/L)	23.170 ± 0.410	19.414 ± 0.615	26.214 ± 0.692	<0.0001
AST (u/L)	22.252 ± 0.285	21.506 ± 0.598	22.857 ± 0.503	0.176
CRP	3.819 ± 0.188	2.515 ± 0.215	4.875 ± 0.226	<0.0001
Minutes sedentary activity	356.817 ± 7.438	350.947 ± 8.859	361.574 ± 8.533	0.259
Alcohol (g)	11.471 ± 0.551	11.315 ± 0.887	11.597 ± 0.816	0.831
HEI-2015	52.543 ± 0.687	54.099 ± 0.938	51.282 ± 0.631	0.002
TC (mmol/L)	4.931 ± 0.045	4.856 ± 0.050	4.991 ± 0.053	0.028
TG (mmol/L)	1.609 ± 0.037	1.222 ± 0.028	1.922 ± 0.042	<0.0001
LDL (mmol/L)	2.897 ± 0.046	2.792 ± 0.046	2.984 ± 0.063	0.01
HDL (mmol/L)	1.388 ± 0.012	1.524 ± 0.013	1.277 ± 0.014	<0.0001
Race (%)				0.002
Mexican American	7.940(0.014)	5.536(1.118)	9.889(1.832)	
Other Hispanic	3.085(0.004)	2.762(0.568)	3.347(0.383)	
Non-Hispanic white	65.371(0.037)	66.687(2.956)	64.305(2.639)	
Non-Hispanic black	14.304(0.016)	16.208(1.849)	12.761(1.629)	
Other races	9.300(0.010)	8.807(1.191)	9.698(1.241)	
Education (%)				0.003
<11th grade	9.006(0.007)	7.990(0.908)	9.830(0.718)	
High school graduate	26.781(0.019)	25.440(2.502)	27.869(1.177)	
Some college	31.165(0.015)	28.381(2.258)	33.421(1.546)	
College graduate or above	33.048(0.030)	38.189(3.656)	28.881(2.408)	
Smoking (%)				0.063
No	84.430(0.026)	83.783(1.570)	84.954(1.112)	
Some day	3.582(0.004)	2.873(0.610)	4.156(0.536)	
Every day	11.989(0.010)	13.344(1.264)	10.890(0.950)	
Dietary supplements				0.156
Yes	58.598(0.025)	57.190(2.121)	59.760(1.722)	
Hypertension (%)				< 0.0001
Yes	39.692(0.023)	23.361(1.839)	52.927(1.984)	
Diabetes (%)				< 0.0001
Yes	15.414(0.008)	4.390(0.669)	24.347(1.228)	

### 3.2. The relationship of log vitamin K and MAFLD

**Figure 1:**
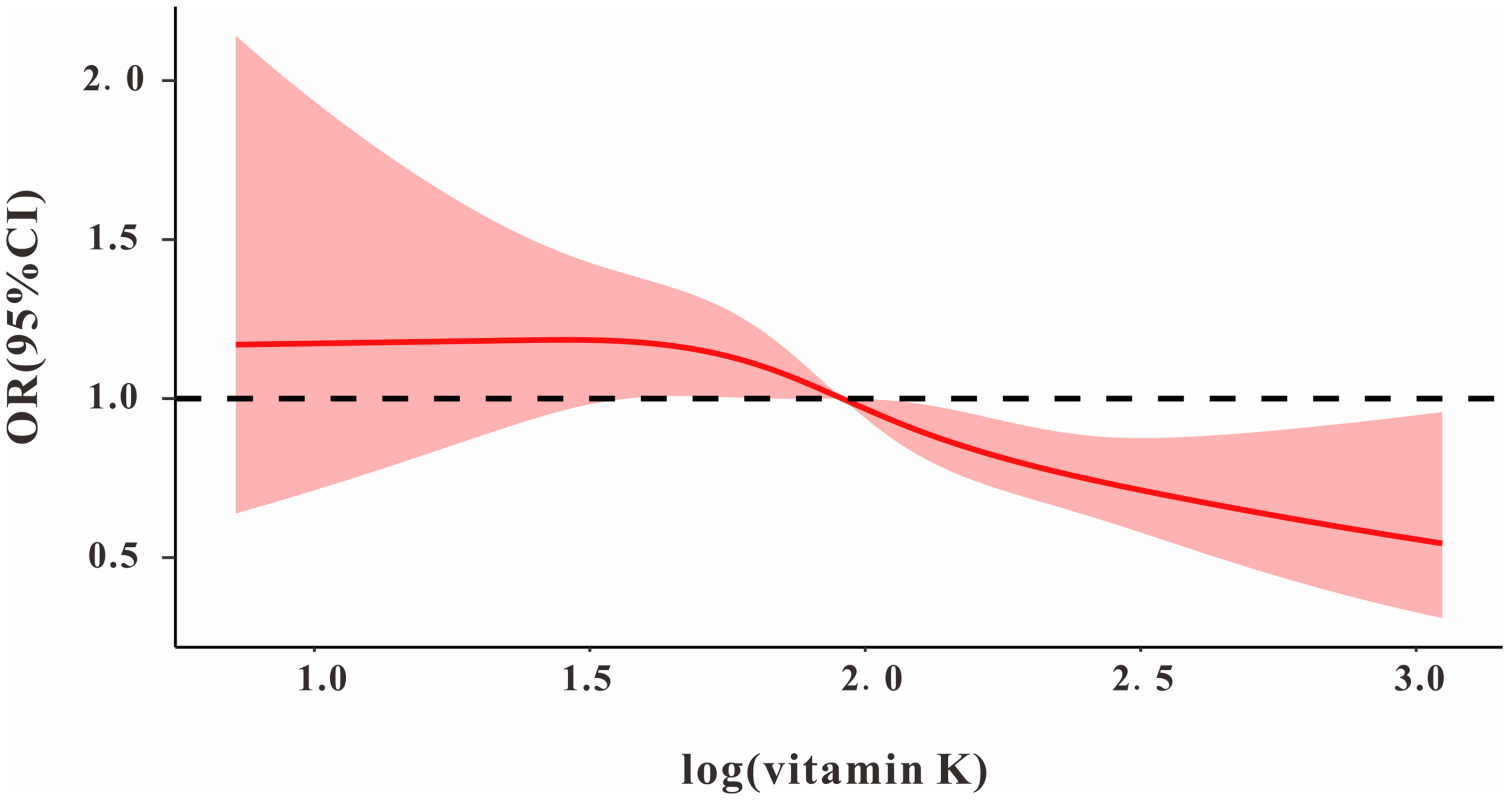
The dose–response relationship between log vitamin K and MAFLD. Model adjusted for age, sex, race, body mass index, education, smoking, alcohol, aspartate aminotransferase, alanine Aminotransferase, minutes sedentary activity, energy, HEI-2015; diabetes mellitus; hypertension, and drug.

We used restricted cubic splines to estimate the dose–response relationship between log vitamin K intake and MAFLD. The results showed a nonlinear relationship between log vitamin K and MAFLD (Figure 1). The log vitamin K inflection point was approximately 2.00 (equal to vitamin K intake of 100 μg), and log vitamin K was a protective factor for MAFLD after the inflection. In a continuous log vitamin K variable, log vitamin K was negatively correlated with MAFLD (OR = 0.462, 95% CI: 0.277–0.796, *p* = 0.006) after adjusting for other control factors in the multivariate regression analysis. Compared with the Q1 quartile of the log vitamin K population, the Q4 quartile had a significantly different MAFLD prevalence in model 3 (OR = 0.488, 95% CI: 0.302–0.787, p = 0.006). *p*-values for trends were significant in all three models (Table 2). Furthermore, as shown in Supplementary Tables S1, S2, TG was negatively correlated with log vitamin K (β = −0.027, 95% CI: −0.041– −0.013, *p* < 0.001) and positively correlated with MAFLD (OR = 2.384, 95%CI: 2.392–3.367, *p* < 0.001); conversely, HDL was positively correlated with log vitamin K (β = 0.132, 95% CI: 0.082–0.182, *p* < 0.001) and negative correlated with MAFLD (OR = 0.164, 95%CI: 0.115–0.232, *p* < 0.001).

**Table 2:** The relationship of log vitamin K and MAFLD.

Exposure	Model 1^a^	Model 2^b^	Model 3^c^
Log vitamin K (continuous)	0.663(0.481,0.915) 0.016	0.559(0.410,0.762) 0.003	0.462(0.277,0.769) 0.006
**Quartile of log vitamin K**			
Q1(≤1.75)	1.000	1.000	1.000
Q2(1.75–1.97)	1.052(0.828,1.337) 0.650	0.978(0.739,1.295) 0.853	0.692(0.515,0.928) 0.017
Q3(1.97–2.21)	0.863(0.665,1.120) 0.243	0.698(0.517,0.941) 0.026	0.592(0.407,0.861) 0.009
Q4(>2.21)	0.708(0.490,1.022) 0.063	0.601(0.415,0.870) 0.015	0.488(0.302,0.787) 0.006
P-trend	0.024	0.004	0.008

a Non adjusted model.

b Minimally adjusted model: adjusted for age, sex, and race.

c Fully adjusted model: age, sex, race, body mass index, education, smoking, alcohol, aspartate aminotransferase, alanine Aminotransferase, minutes sedentary activity, energy, HEI-2015; diabetes mellitus; hypertension, and drug.

### 3.3. Subgroup analysis

We divided the population into those with and without dietary supplements. In those who did not take dietary supplements, we found that log vitamin K was a protective factor for MAFLD, regardless of the adjustment for other covariates (*p* < 0.05). In model 3, compared with the Q1 (≤1.75, equal to <56 μg), the Q4 (>2.21, equal to >162 μg) population had a lower risk of MAFLD (OR = 0.373, 95% CI: 0.186–0.751, *p* = 0.009). Among those who consumed dietary supplements, although log vitamin K was found to be a protective factor for MAFLD in model 1 and model 2 (p < 0.05), the relationship between log vitamin K and MAFLD was not statistically significant in model 3 (OR = 0.489, 95% CI: 0.238–1.001, *p* = 0.050). The details are given in Table 3.

**Table 3:** The relationship of log vitamin K and MAFLD in with or without dietary supplements.

Population	Exposure	Model 1^a^	Model 2^b^	Model 3^c^
Without dietary supplements	Log vitamin K (continuous)	0.709(0.496,1.012) 0.057	0.632(0.425,0.938) 0.028	0.346(0.159,0.750) 0.011
	**Quartile of log vitamin K**			
	Q1(≤1.75)	1.000	1.000	1.000
	Q2(1.75–1.97)	1.273(0.802,2.022) 0.277	1.192(0.652,2.182) 0.503	0.962(0.504,1.837) 0.901
	Q3(1.97–2.21)	0.938(0.648,1.356) 0.710	0.812(0.511,1.289) 0.302	0.721(0.420,1.239) 0.217
	Q4(>2.21)	0.714(0.508,1.004) 0.052	0.658(0.434,0.996) 0.048	0.373(0.186,0.751) 0.009
	P-trend	0.083	0.035	0.018
With dietary supplements	Log vitamin K (continuous)	0.569(0.329,0.986) 0.045	0.509(0.286,0.906) 0.027	0.555(0.247,1.247) 0.142
	**Quartile of log vitamin K**			
	Q1(≤1.75)	1.000	1.000	1.000
	Q2(1.75–1.97)	0.816(0.598,1.115) 0.181	0.770(0.526,1.127) 0.144	0.500(0.300,0.833) 0.011
	Q3(1.97–2.21)	0.717(0.467,1.101) 0.117	0.585(0.357,0.959) 0.038	0.519(0.286,0.940) 0.033
	Q4(>2.21)	0.587(0.348,0.989) 0.046	0.517(0.297,0.902) 0.027	0.489(0.238,1.001) 0.050
	P-trend	0.059	0.030	0.142

a Non adjusted model.

b Minimally adjusted model: adjusted for age, sex, and race.

c Fully adjusted model: age, sex, race, body mass index, education, smoking, alcohol, aspartate aminotransferase, alanine Aminotransferase, minutes sedentary activity, energy, HEI-2015; diabetes mellitus; hypertension, and drug.

We also analyzed other subgroups and found that in the population aged <40 or 40–65 years, men and women, BMI < 30 kg/m^2^, HEI-2015 ≤ 50 or > 58, energy ≤1,641 or > 2,358 kcal, non-DM or DM, and non-hypertension, the log vitamin K was negatively associated with the prevalence of MAFLD (*p* < 0.05). These potential factors did not interact with log vitamin K values in the prevalence of MAFLD. The details are provided in Supplementary Figure S2.

### 3.4. Sensitivity analysis

We performed a series of sensitivity analyses to assess the robustness of the findings. Collinearity diagnostics showed that vitamin K did not have collinearity with other variables (all VIF < 10) in multivariate regression models, regardless of sex (Supplementary Table S3). After replacing the extremes of vitamin K intake, the correlation between log vitamin K and MAFLD in models 1, 2, and 3 was consistent with the findings in Table 2, showing good model stability in this study (Supplementary Table S4).

## 4. Discussion

Studies have shown that vitamins are essential trace elements involved in various biological functions, and the processes of their metabolism, storage, and activation occur in the liver (28); therefore, liver diseases are usually associated with vitamin disorders. Metabolic fatty liver disease is characterized by hepatic steatosis combined with one or more metabolic abnormalities. In this study, fatty liver degeneration was defined by CAP. Compared with conventional ultrasound, CAP can detect a milder degree of steatosis and has a good correlation with liver biopsy being used for steatosis detection (6). Although the number of patients diagnosed with MAFLD is similar to the number of patients with NAFLD (29), the risk of cirrhosis and liver cancer is more likely to be higher in people with MAFLD because they have higher liver enzyme levels and more diseases—related to glucose and lipid metabolism (30). To the best of our knowledge, this is the first study to explore the relationship between vitamin K intake from diet and dietary supplements and MAFLD. The results showed that compared with the non-MAFLD population, vitamin K intake was lower in the MAFLD population (123.113 ± 4.635 μg vs. 145.064 ± 7.759 μg). Higher vitamin K intake could lower the risk of MAFLD, compared to the population with a vitamin K intake of ≤56.23 μg (equal to log vitamin K ≤ 1.75), there was a 50% risk of MAFLD in the population with a vitamin K intake of >162.18 μg (equal to log vitamin K > 2.21), which was 40% in the population without dietary supplements intake.

Vitamin K is a lipophilic micronutrient divided into phylloquinone and menaquinone (31). In humans, the dietary intake of phylloquinone comes mainly from vegetables and vegetable oils, while menaquinone comes from animal products and bacterially fermented foods (32). Vitamin K was first known for its role in blood clotting, but it can also regulate inflammation, and reduce oxidation (33, 34). Ferroptosis is regulated by multiple cellular metabolic events, including redox homeostasis, iron handling, mitochondrial activity, and metabolism of amino acids, lipids, and sugars (35). Recent studies have found that the vitamin K cycle can protect cells against detrimental lipid peroxidation and ferroptosis (36). In animal experiments, it was found that vitamin K supplementation can downregulate the glycosylated blood glucose protein and insulin resistance in diabetic rats (37), and reduce body fat accumulation and serum triglyceride (TRG) levels in obese rats (38). Population trials have found that increased dietary intake of vitamin K appears to be associated with a lower incidence of DM (39, 40). Vitamin K administration in mice fed a high-fat diet can significantly increase Gla-Gas6 protein levels, upregulate AMPK phosphorylation state, and reduce SREBP1 and PPARα expression. Therefore, it is speculated that the mechanism of vitamin K on lipid metabolism may be mediated by the activation of Gas6 protein (41), then regulating AMPK SREBP1/FAS and PPARα/CPT1A/UCP2 signaling cascades in hepatic lipid metabolism, thereby maintaining whole-body lipid homeostasis (42). Vitamin K is involved in the activation of the AMP-activated protein kinase/sirtuin 1 pathway in the liver, which, in turn, upregulates phosphoinositide 3-kinase and glucose transporter 2 to reduce insulin resistance and fasting blood glucose (37). In this study, we also found that higher vitamin K intake could reduce the risk of MAFLD in the DM population, and individuals with a higher intake of vitamin K had lower TG and higher HDL levels. These results suggest that the anti-inflammatory, anti-ferroptosis, and antioxidant effects of vitamin K regulate glucose and lipid metabolism disorders (43). The pathogenesis of MAFLD is mainly related to metabolic disorders and altered glucose-insulin homeostasis; therefore, vitamin K may reduce the risk of MAFLD by stabilizing glucose-lipid metabolism.

Interestingly, in the stratified analysis, when adjusting for no other variables or adjusting for sex, age, and race, vitamin K significantly reduced the risk of MAFLD among those who consumed dietary supplements, but when the model continued to add moderator variables (activity, other nutrients, and disease), this protective effect was not statistically significant. This result may indicate that the effect of vitamin K on MAFLD may not be significant in the population consuming dietary supplements, which may be affected by other dietary nutrients. In this study, we also observed inconsistency regarding the relationship between vitamin K and MAFLD based on the HEI-2015 and energy subgroup analyses. In another study, a diet rich in vitamin K was associated with a lower prevalence of decreased high-density lipoprotein cholesterol, high serum triglycerides, and hyperglycemia. However, after adjusting for dietary confounders, the effect remained significant only for hyperglycemia (44). Studies have shown that vitamin K function is also related to age, sex, and/or menopause. Additionally, since vitamin K is a fat-soluble vitamin, obesity increases the storage of some fat-soluble nutrients (19). Therefore, vitamin K intake may not play a role in obesity. Age, sex, and menopause have been shown to affect vitamin K metabolism (31). Plasma phylloquinone levels were found to be significantly higher in the elderly (>60 years) than in the young (<40 years) independent of dietary intake (45, 46). This finding indicates that there may be a protective effect of vitamin K against MAFLD in young men, but not in older age. In this study, it was also found that vitamin K did not reduce the risk of MAFLD in the elderly and obese groups. In short, although it is unclear how vitamin K exerts its antioxidant and anti-inflammatory effects, this study suggests that increasing vitamin K intake may reduce the risk of MAFLD, especially in populations that do not consume any dietary supplements.

Our study has several strengths. First, the source of vitamin K is not only food but also dietary supplements. The relationship between vitamin K and MAFLD was analyzed between those who used dietary supplements and those who did not. Second, the NHANES database contains a large and nationally representative sample of the adult population in the United States. Third, the study used the weight in the analysis method to adjust for covariates in different models and considered the association of vitamin K and MAFLD in different populations, thus performing a sensitivity analysis. However, our study also had a few limitations. First, the cross-sectional design of our study could not accurately reflect the causal relationship between vitamin K levels and the risk of MAFLD. Second, 24 h dietary interviews do not necessarily reflect long-term dietary consumption habits. Third, although the study was adjusted for many covariates, the effect of unmeasured confounders cannot be ruled out. Fourth, dietary supplements are not intended for people who only take vitamin K supplements and do not distinguish between different food sources of vitamins and different vitamin subtypes (K1 and K2).

## 5. Conclusion

Vitamin K is inversely associated with the prevalence of MAFLD, suggesting that a higher vitamin K intake is associated with a lower risk of MAFLD. This negative relationship is more significant in the individuals who were not taking dietary supplements. Nevertheless, this study was an observational study, and the plasma concentration of vitamin K was not detected to confirm the effect of the vitamin K increase in the body. Therefore, the relationship between vitamin K intake and plasma vitamin K levels and the relationship between plasma vitamin K and glucose and lipid metabolism should be continued explored in population studies, and their possible mechanisms should be explored in animal experiments.

## Data availability statement

The original contributions presented in the study are included in the article/Supplementary material, further inquiries can be directed to the corresponding authors.

## Ethics statement

The Ethics Review Board of the National Center for Health Statistics approved the NHANES protocol and informed consent was obtained from all participants (18). All data can be freely downloaded from NHANES website (https://www.cdc.gov/nchs/nhanes/index.htm).

## Author contributions

XW, WZ, JH, HL, and JG contributed to the study conception and design. XW, HL, and JG designed the study. WZ and JH organized and analyzed the data and wrote the manuscript. XW and HL contributed materials/analysis tools. All authors contributed to the article and approved the submitted version.

## Funding

This study was supported by the Incubation Fund of Zhongshan Hospital (Xiamen), Fudan University (No. 2020ZS XM YS 24).

## Conflict of interest

The authors declare that the research was conducted in the absence of any commercial or financial relationships that could be construed as a potential conflict of interest.

## Publisher’s note

All claims expressed in this article are solely those of the authors and do not necessarily represent those of their affiliated organizations, or those of the publisher, the editors and the reviewers. Any product that may be evaluated in this article, or claim that may be made by its manufacturer, is not guaranteed or endorsed by the publisher.

## Footnotes

^1^https://www.cdc.gov/nchs/nhanes/index.htm

## Abbreviations

MFALD: Metabolic dysfunction-associated fatty liver disease
NAFLD: Non-alcoholic fatty liver disease
NHANES: National Health and Nutrition Examination Surveys
DGA: Dietary Guidelines for Americans
CDC: Centers for Disease Control and Prevention
HEI: Healthy Eating index
CRP: C-reactive protein
AST: Aspartate aminotransferase
ALT: Alanine aminotransferase
SE: Standard error
BMI: body mass index
LSM: Liver stiffness measurements
DM: Diabetes mellitus
TC: Cholesterol
TG: Triglycerides
LDL: Low-density lipoprotein
HDL: High-density lipoprotein

## References

[1] Jennings J Faselis C Yao MD. Nafld-Nash: an under-recognized epidemic. Curr Vasc Pharmacol. (2018) 16:209–13. doi: 10.2174/1570161115666170622074007, PMID: 28676024

[2] Chalasani N Younossi Z Lavine JE Charlton M Cusi K Rinella M. The diagnosis and Management of Nonalcoholic Fatty Liver Disease: practice guidance from the American Association for the Study of Liver Diseases. Hepatology. (2018) 67:328–57. doi: 10.1002/hep.29367, PMID: 28714183

[3] Saeed A Dullaart RPF Schreuder T Blokzijl H Faber KN. Disturbed vitamin a metabolism in non-alcoholic fatty liver disease (Nafld). Nutrients. (2017) 10:29. doi: 10.3390/nu10010029, PMID: 29286303 PMC5793257

[4] Wong VW Lazarus JV. Prognosis of Mafld Vs. Nafld and implications for a nomenclature change. J Hepatol. (2021) 75:1267–70. doi: 10.1016/j.jhep.2021.08.020, PMID: 34464658

[5] Valencia-Rodríguez A Vera-Barajas A Chávez-Tapia NC Uribe M Méndez-Sánchez N. Looking into a new era for the approach of metabolic (dysfunction) associated fatty liver disease. Ann Hepatol. (2020) 19:227–9. doi: 10.1016/j.aohep.2020.04.001, PMID: 32359519

[6] Ferraioli G Soares Monteiro LB. Ultrasound-based techniques for the diagnosis of liver Steatosis. World J Gastroenterol. (2019) 25:6053–62. doi: 10.3748/wjg.v25.i40.6053, PMID: 31686762 PMC6824276

[7] Papatheodoridi M Cholongitas E. Diagnosis of non-alcoholic fatty liver disease (Nafld): current concepts. Curr Pharm Des. (2018) 24:4574–86. doi: 10.2174/1381612825666190117102111 30652642

[8] Pierantonelli I Svegliati-Baroni G. Nonalcoholic fatty liver disease: basic Pathogenetic mechanisms in the progression from Nafld to Nash. Transplantation. (2019) 103:e1–e13. doi: 10.1097/tp.0000000000002480, PMID: 30300287

[9] Manne V Handa P Kowdley KV. Pathophysiology of nonalcoholic fatty liver disease/nonalcoholic Steatohepatitis. Clin Liver Dis. (2018) 22:23–37. doi: 10.1016/j.cld.2017.08.007 29128059

[10] Fotbolcu H Zorlu E. Nonalcoholic fatty liver disease as a multi-systemic disease. World J Gastroenterol. (2016) 22:4079–90. doi: 10.3748/wjg.v22.i16.4079, PMID: 27122660 PMC4837427

[11] Sumida Y Yoneda M. Current and future pharmacological therapies for Nafld/Nash. J Gastroenterol. (2018) 53:362–76. doi: 10.1007/s00535-017-1415-1, PMID: 29247356 PMC5847174

[12] Federico A Dallio M Masarone M Gravina AG Di Sarno R Tuccillo C. Evaluation of the effect derived from Silybin with vitamin D and vitamin E administration on clinical, metabolic, endothelial dysfunction, oxidative stress parameters, and serological worsening markers in nonalcoholic fatty liver disease patients. Oxidative Med Cell Longev. (2019) 2019:8742075–12. doi: 10.1155/2019/8742075, PMID: 31737175 PMC6815609

[13] Nagashimada M Ota T. Role of vitamin E in nonalcoholic fatty liver disease. IUBMB Life. (2019) 71:516–22. doi: 10.1002/iub.1991 30592129

[14] Zhang Z Thorne JL Moore JB. Vitamin D and nonalcoholic fatty liver disease. Curr Opin Clin Nutr Metab Care. (2019) 22:449–58. doi: 10.1097/mco.0000000000000605 31589177

[15] Dowd P Ham SW Naganathan S Hershline R. The mechanism of action of vitamin K. Annu Rev Nutr. (1995) 15:419–40. doi: 10.1146/annurev.nu.15.070195.002223 8527228

[16] Ferland G. The discovery of vitamin K and its clinical applications. Ann Nutr Metab. (2012) 61:213–8. doi: 10.1159/000343108, PMID: 23183291

[17] Lai Y Masatoshi H Ma Y Guo Y Zhang B. Role of vitamin K in intestinal health. Front Immunol. (2021) 12:791565. doi: 10.3389/fimmu.2021.791565, PMID: 35069573 PMC8769504

[18] Liu Y Mosenthin R Zhao L Zhang J Ji C Ma Q. Vitamin K alleviates bone calcium loss caused by salmonella Enteritidis through carboxylation of Osteocalcin. J Anim Sci Biotechnol. (2021) 12:80. doi: 10.1186/s40104-021-00604-z, PMID: 34253252 PMC8276384

[19] Shea MK Booth SL Gundberg CM Peterson JW Waddell C Dawson-Hughes B. Adulthood obesity is positively associated with adipose tissue concentrations of vitamin K and inversely associated with circulating indicators of vitamin K status in men and women. J Nutr. (2010) 140:1029–34. doi: 10.3945/jn.109.118380, PMID: 20237066 PMC2855266

[20] Ciardullo S Monti T Perseghin G. Prevalence of liver Steatosis and fibrosis detected by transient Elastography in adolescents in the 2017-2018 National Health and nutrition examination survey. Clin Gastroenterol Hepatol. (2021) 19:384–90.e1. doi: 10.1016/j.cgh.2020.06.048, PMID: 32623006

[21] Weng Z Ou W Huang J Singh M Wang M Zhu Y. Circadian misalignment rather than sleep duration is associated with Mafld: a population-based propensity score-matched study. Nat Sci Sleep. (2021) 13:103–11. doi: 10.2147/nss.S290465, PMID: 33542668 PMC7853435

[22] Steinfeldt LC Martin CL Clemens JC Moshfegh AJ. Comparing two days of dietary intake in what we eat in America (Wweia), Nhanes, 2013-2016. Nutrients. (2021) 13:2621. doi: 10.3390/nu13082621 34444781 PMC8399790

[23] Cassinotto C Boursier J de Lédinghen V Lebigot J Lapuyade B Cales P. Liver stiffness in nonalcoholic fatty liver disease: a comparison of supersonic shear imaging, Fibroscan, and Arfi with liver biopsy. Hepatology. (2016) 63:1817–27. doi: 10.1002/hep.28394, PMID: 26659452

[24] Westgard JO Barry PL Hunt MR Groth T. A multi-rule Shewhart chart for quality control in clinical chemistry. Clin Chem. (1981) 27:493–501. doi: 10.1093/clinchem/27.3.493, PMID: 7471403

[25] Krebs-Smith SM Pannucci TE Subar AF Kirkpatrick SI Lerman JL Tooze JA. Update of the healthy eating index: Hei-2015. J Acad Nutr Diet. (2018) 118:1591–602. doi: 10.1016/j.jand.2018.05.021, PMID: 30146071 PMC6719291

[26] National Health and Nutrition Examination Survey. NHANES Demographics Data. Available at: https://wwwn.cdc.gov/nchs/nhanes

[27] Centers for Disease Control and Prevention (CDC), National Center for Health Statistics (NCHS). National Health and Nutrition Examination Survey 2017–March 2020 Prepandemic File: Sample Design, Estimation, and Analytic Guidelines. (2022). Available at: https://wwwn.cdc.gov/nchs/nhanes/analyticguidelines.aspx 35593699

[28] Bjelakovic G Nikolova D Bjelakovic M Gluud C. Vitamin D supplementation for chronic liver diseases in adults. Cochrane Database Syst Rev. (2017) 11:CD011564. doi: 10.1002/14651858.CD011564.pub2, PMID: 29099543 PMC6485973

[29] Ciardullo S Perseghin G. Prevalence of Nafld, Mafld and associated advanced fibrosis in the contemporary United States population. Liver Int. (2021) 41:1290–3. doi: 10.1111/liv.14828, PMID: 33590934

[30] Lin S Huang J Wang M Kumar R Liu Y Liu S. Comparison of Mafld and Nafld diagnostic criteria in real world. Liver Int. (2020) 40:2082–9. doi: 10.1111/liv.14548, PMID: 32478487

[31] Booth SL Suttie JW. Dietary intake and adequacy of vitamin K. J Nutr. (1998) 128:785–8. doi: 10.1093/jn/128.5.785 9566982

[32] Schurgers LJ Vermeer C. Determination of Phylloquinone and Menaquinones in food. Effect of food matrix on circulating vitamin K concentrations. Haemostasis. (2000) 30:298–307. doi: 10.1159/000054147, PMID: 11356998

[33] Canfield LM Davy LA Thomas GL. Anti-oxidant/pro-oxidant reactions of vitamin K. Biochem Biophys Res Commun. (1985) 128:211–9. doi: 10.1016/0006-291x(85)91666-3, PMID: 3985964

[34] Price PA Rice JS Williamson MK. Conserved phosphorylation of Serines in the Ser-X-Glu/Ser(P) sequences of the vitamin K-dependent matrix Gla protein from shark, lamb, rat, cow, and human. Protein Sci. (1994) 3:822–30. doi: 10.1002/pro.5560030511, PMID: 8061611 PMC2142713

[35] Jiang X Stockwell BR Conrad M. Ferroptosis: mechanisms, biology and role in disease. Nat Rev Mol Cell Biol. (2021) 22:266–82. doi: 10.1038/s41580-020-00324-8, PMID: 33495651 PMC8142022

[36] Mishima E Ito J Wu Z Nakamura T Wahida A Doll S. A non-canonical vitamin K cycle is a potent Ferroptosis suppressor. Nature. (2022) 608:778–83. doi: 10.1038/s41586-022-05022-3, PMID: 35922516 PMC9402432

[37] Dihingia A Ozah D Ghosh S Sarkar A Baruah PK Kalita J. Vitamin K1 inversely correlates with Glycemia and insulin resistance in patients with type 2 diabetes (T2d) and positively regulates Sirt1/Ampk pathway of glucose metabolism in liver of T2d mice and hepatocytes cultured in high glucose. J Nutr Biochem. (2018) 52:103–14. doi: 10.1016/j.jnutbio.2017.09.022, PMID: 29175667

[38] Sogabe N Maruyama R Baba O Hosoi T Goseki-Sone M. Effects of long-term vitamin K(1) (Phylloquinone) or vitamin K(2) (Menaquinone-4) supplementation on body composition and serum parameters in rats. Bone. (2011) 48:1036–42. doi: 10.1016/j.bone.2011.01.020, PMID: 21295170

[39] Ibarrola-Jurado N Salas-Salvadó J Martínez-González MA Bulló M. Dietary Phylloquinone intake and risk of type 2 diabetes in elderly subjects at high risk of cardiovascular disease. Am J Clin Nutr. (2012) 96:1113–8. doi: 10.3945/ajcn.111.033498, PMID: 23034962

[40] Juanola-Falgarona M Salas-Salvadó J Estruch R Portillo MP Casas R Miranda J. Association between dietary Phylloquinone intake and peripheral metabolic risk markers related to insulin resistance and diabetes in elderly subjects at high cardiovascular risk. Cardiovasc Diabetol. (2013) 12:7. doi: 10.1186/1475-2840-12-7, PMID: 23298335 PMC3558443

[41] Bellido-Martín L de Frutos PG. Vitamin K-dependent actions of Gas6. Vitam Horm. (2008) 78:185–209. doi: 10.1016/s0083-6729(07)00009-x, PMID: 18374195

[42] Bordoloi J Ozah D Bora T Kalita J Manna P. Gamma-Glutamyl Carboxylated Gas6 mediates the beneficial effect of vitamin K on lowering hyperlipidemia via regulating the Ampk/Srebp1/Pparα signaling Cascade of lipid metabolism. J Nutr Biochem. (2019) 70:174–84. doi: 10.1016/j.jnutbio.2019.05.006, PMID: 31226525

[43] Shearer MJ Okano T. Key pathways and regulators of vitamin K function and intermediary metabolism. Annu Rev Nutr. (2018) 38:127–51. doi: 10.1146/annurev-nutr-082117-051741, PMID: 29856932

[44] Knapen MHJ Jardon KM Vermeer C. Vitamin K-induced effects on body fat and weight: results from a 3-year vitamin K2 intervention study. Eur J Clin Nutr. (2018) 72:136–41. doi: 10.1038/ejcn.2017.146, PMID: 28952607

[45] Bach AU Anderson SA Foley AL Williams EC Suttie JW. Assessment of vitamin K status in human subjects administered "Minidose" warfarin. Am J Clin Nutr. (1996) 64:894–902. doi: 10.1093/ajcn/64.6.894, PMID: 8942414

[46] Ferland G Sadowski JA O'Brien ME. Dietary induced subclinical vitamin K deficiency in Normal human subjects. J Clin Invest. (1993) 91:1761–8. doi: 10.1172/jci116386, PMID: 8473516 PMC288156

