# Supplementary material for: The relationship between vitamin K and metabolic dysfunction-associated fatty liver disease among the United States population: National Health and Nutrition Examination Survey 2017–2018
Source: Front Nutr. 2023 Apr 14;10:1086477. doi: 10.3389/fnut.2023.1086477 (PMC10249661; doi:10.3389/fnut.2023.1086477)
Supplement: Supplementary file 1 [file Data_Sheet_1.docx]

Supplement materials

Table S1 The relationship of log vitamin K and blood lipids

| Variable | Estimate | 95%CI | P-value |
| --- | --- | --- | --- |
| TC (mmol/l) | -0.003 | (-0.021,0.014) | 0.689 |
| TG (mmol/l) | -0.027 | (-0.041, -0.013) | <0.001 |
| LDL (mmol/l) | -0.026 | (-0.058,0.007) | 0.111 |
| HDL (mmol/l) | 0.132 | (0.082,0.182) | <0.0001 |

TC: Cholesterol; TG: Triglycerides; LDL: Low-density lipoprotein; HDL: High-density lipoprotein

Table S2 The relationship of blood lipids and MAFLD

| Variable | OR | 95%CI | P-value |
| --- | --- | --- | --- |
| TC (mmol/l) | 1.135 | （1.012,1.273） | 0.032 |
| TG (mmol/l) | 2.838 | (2.392,3.367) | <0.0001 |
| LDL (mmol/l) | 1.264 | (1.054,1.515) | 0.015 |
| HDL (mmol/l) | 0.164 | (0.115,0.232) | <0.0001 |

TC: Cholesterol; TG: Triglycerides; LDL: Low-density lipoprotein; HDL: High-density lipoprotein

Table S3 Collinearity of the diagnosis between the variables

| Variable | Statistical tolerance | VIF |
| --- | --- | --- |
| Age (y) | 0.611 | 1.638 |
| Education | 0.801 | 1.248 |
| Race | 0.877 | 1.140 |
| Log vitamin K | 0.689 | 1.451 |
| Energy (kcal) | 0.666 | 1.501 |
| BMI (kg/m^2^) | 0.789 | 1.267 |
| ALT(u/l) | 0.353 | 2.830 |
| AST(u/l) | 0.372 | 2.688 |
| CRP | 0.892 | 1.121 |
| Minutes sedentary activity | 0.927 | 1.079 |
| Alcohol (g) | 0.878 | 1.139 |
| HEI-2015 | 0.749 | 1.334 |
| Smoking | 0.891 | 1.123 |
| dietary supplements | 0.834 | 1.200 |
| Hypertension | 0.704 | 1.421 |
| Diabetes | 0.819 | 1.221 |

Table S4 Association of log vitamin K with MAFLD (after deal with extreme vitamin K)

| Exposure | Model 1^a^ | Model 2^b^ | Model 3^c^ |
| --- | --- | --- | --- |
| Log vitamin K (continuous) | 0.659(0.472,0.919) 0.018 | 0.550(0.399,0.758) 0.003 | 0.456(0.271,0.767) 0.006 |
| Quartile of log vitamin K |  |  |  |
| Q1(≤1.75) | 1.000 | 1.000 | 1.000 |
| Q2(1.75-1.97) | 1.052(0.828,1.337) 0.650 | 0.978(0.739,1.295) 0.853 | 0.692(0.515,0.928) 0.017 |
| Q3(1.97-2.21) | 0.863(0.665,1.120) 0.243 | 0.698(0.517,0.941) 0.026 | 0.592(0.407,0.861) 0.009 |
| Q4(>2.21) | 0.708(0.490,1.022) 0.063 | 0.601(0.415,0.870) 0.015 | 0.488(0.302,0.787) 0.006 |
| P-trend | 0.024 | 0.004 | 0.009 |

^a^ Non adjusted model

^b^ Minimally adjusted model: adjusted for age，sex, and race

^c^ Fully adjusted model: age, sex, race, body mass index, education, smoking, alcohol, aspartate aminotransferase, alanine Aminotransferase, minutes sedentary activity, energy, HEI-2015; diabetes mellitus; hypertension, and drug

**
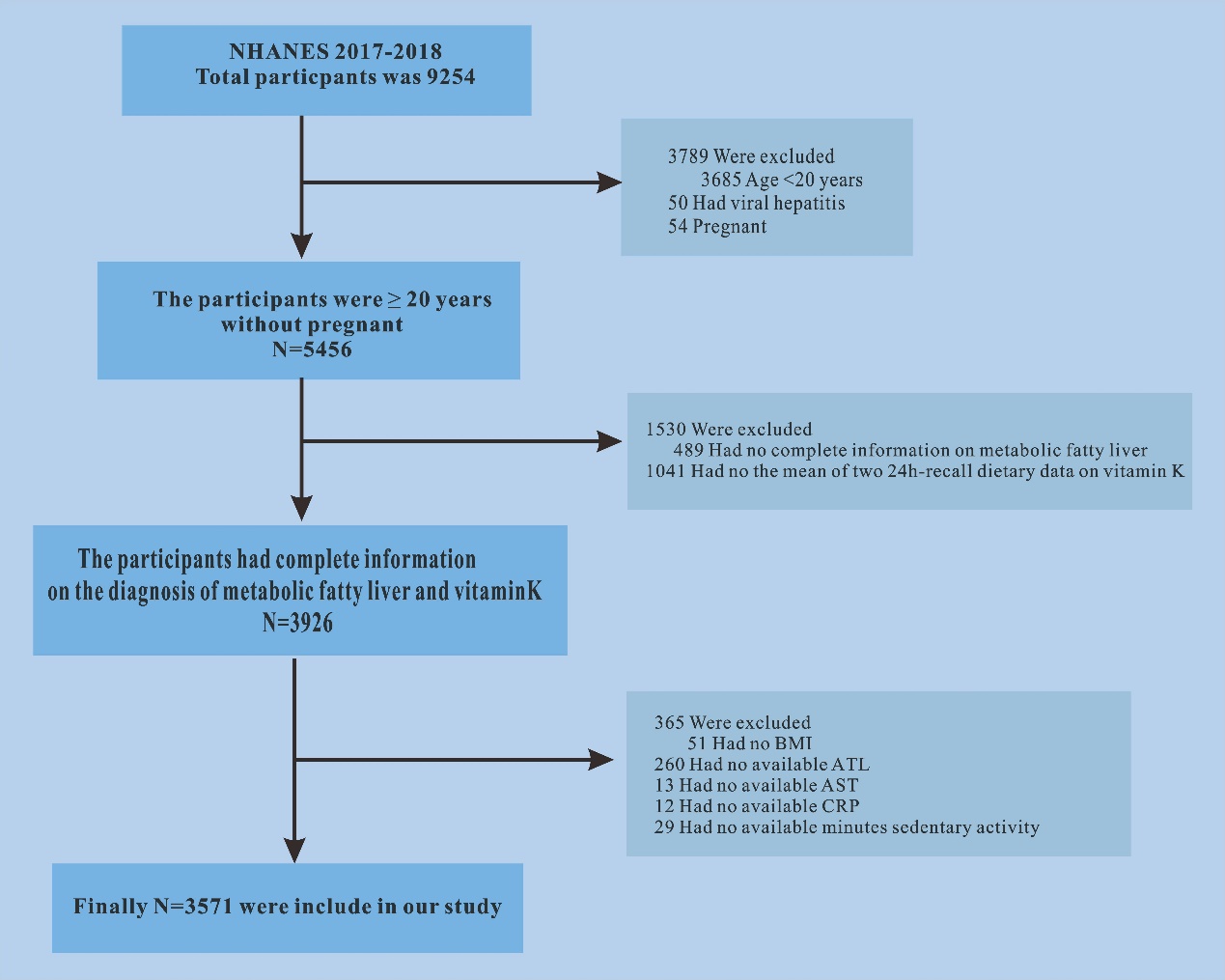
**

**FigS1 The flowchart of study participants**

**
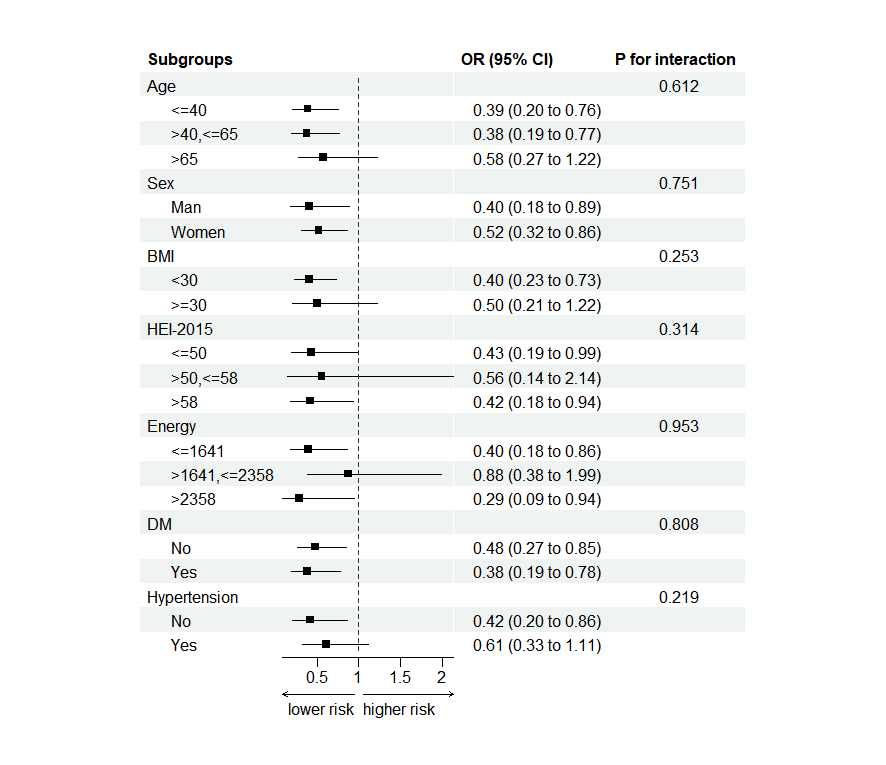
**

**Figure S2** The subgroup analysis and interaction of log vitamin K and MAFLD The model was adjusted for age, race, sex, body mass index, education, smoking, drinking, aspartate aminotransferase, alanine aminotransferase, minutes sedentary activity, energy, HEI-2015, diabetes mellitus, hypertension, and drug.
